# Interphase Chromosomes in Replicative Senescence: Chromosome Positioning as a Senescence Biomarker and the Lack of Nuclear Motor-Driven Chromosome Repositioning in Senescent Cells

**DOI:** 10.3389/fcell.2021.640200

**Published:** 2021-05-24

**Authors:** Ishita S. Mehta, Kumars Riyahi, Rita Torres Pereira, Karen J. Meaburn, Martin Figgitt, Ian R. Kill, Christopher H. Eskiw, Joanna M. Bridger

**Affiliations:** ^1^Centre for Genome Engineering and Maintenance, Division of Biosciences, Department of Life Sciences, College of Health, Medicine and Life Sciences, Kingston Lane, Brunel University London, Uxbridge, United Kingdom; ^2^Tata Institute of Fundamental Research, Mumbai, India; ^3^Department of Life Sciences, Birmingham City University, Birmingham, United Kingdom; ^4^Department of Food and Bioproduct Sciences, University of Saskatchewan, Saskatoon, SK, Canada

**Keywords:** replicative senescence (RS), genome organisation, nuclear motors, chromatin dynamics, chromosome territories, nuclear myosin 1β, chromosome 10

## Abstract

This study demonstrates, and confirms, that chromosome territory positioning is altered in primary senescent human dermal fibroblasts (HDFs). The chromosome territory positioning pattern is very similar to that found in HDFs made quiescent either by serum starvation or confluence; but not completely. A few chromosomes are found in different locations. One chromosome in particular stands out, chromosome 10, which is located in an intermediate location in young proliferating HDFs, but is found at the nuclear periphery in quiescent cells and in an opposing location of the nuclear interior in senescent HDFs. We have previously demonstrated that individual chromosome territories can be actively and rapidly relocated, with 15 min, after removal of serum from the culture media. These chromosome relocations require nuclear motor activity through the presence of nuclear myosin 1β (NM1β). We now also demonstrate rapid chromosome movement in HDFs after heat-shock at 42°C. Others have shown that heat shock genes are actively relocated using nuclear motor protein activity via actin or NM1β (Khanna et al., 2014; Pradhan et al., 2020). However, this current study reveals, that in senescent HDFs, chromosomes can no longer be relocated to expected nuclear locations upon these two types of stimuli. This coincides with a entirely different organisation and distribution of NM1β within senescent HDFs.

## Introduction

Senescence is described as a gradual accumulation of non-dividing cells throughout the reproductive life span of culture (Hayflick and Moorhead, 1961; Kill et al., 1994; Ben-Porath and Weinberg, 2004), it is a major obstacle to continuous propagation of cells, and thus is often regarded as a tumour suppressing mechanism (Kill, 1998; Campisi, 2001, 2003a, 2003b). Various studies showing a functional link between increasing number of senescent cells (Dimri et al., 1995; Li et al., 1997; Pawelec et al., 1999) and decreasing activity of stem cells (Collado et al., 2007) with the age of tissue or organism, suggested a link between cellular senescence and organismal ageing (Hayflick and Moorhead, 1961; Campisi, 2003b; Smith and Kipling, 2004; Collado et al., 2007). More recently, organismal ageing has been directly proven to be caused by the accumulation of senescent cells within an organisms’ body (Chang et al., 2016; Folgueras et al., 2018), adding a burden to tissues by secreting a plethora of antagonistic and deleterious molecules (Gorgoulis et al., 2019) through the Secretory Associated Senescence Pathway (SASP), inducing senescence in nearby cells (Acosta et al., 2013), termed paracrine senescence (Hernandez-Segura et al., 2018).

Senescence can be caused by various different stimuli, and the different types of senescent cells may even have different roles within the body (Bridger and Foster, 2021). In replicative senescence (RS), cells reach senescence through serial division, and are permanently arrested although metabolically active (Hayflick and Moorhead, 1961). RS cells display telomere shortening (Harley et al., 1990; Allsopp et al., 1995; Blackburn, 2001; Cawthon et al., 2003; Masutomi et al., 2003; Ben-Porath and Weinberg, 2004; Ogami et al., 2004; Davis and Kipling, 2005; Canela et al., 2007), with accumulation of DNA damage through an inability to repair it (Chen et al., 2020), de-repression of p16*^*INK*4a^* loci (Zindy et al., 1997; Chkhotua et al., 2003; Krishnamurthy et al., 2004; Ressler et al., 2006) and alterations in Rb/p13 or p53/p21^*CIP*1^ pathways, both inducing senescence in different ways (Chen et al., 2020). Oxidative stress-induced premature senescence (SIPS) is elicited through external or internal metabolic oxidative agents, causing severe or irreparable DNA damage (te Poele et al., 2002; d’Adda di Fagagna et al., 2003; Parrinello et al., 2003; Bartkova et al., 2006; Di Micco et al., 2006). Oncogene-induced senescence (OIS) comes about via the activation of oncogenes such as Ras or the inactivation of tumour suppressor genes (Prieur and Peeper, 2008). Senescence can also be induced by mitochondrial dysfunction (Wiley et al., 2016), chemotherapy drugs, inhibition of histone methyl transferases or histone deactylases (Petrova et al., 2016).

Cellular senescence is known to be a mechanism to avoid tumourigenesis but it also has regulatory roles in embryogenesis and wound healing (Coppé et al., 2010; Muñoz-Espín et al., 2013, Storer et al., 2013; Graziano and Gonzalo, 2017). Senescence is such an important mechanism it is evolutionary conserved, cells from mammals, birds, reptiles, flies, and yeast undergo growth arrest and exhibit senescent phenotypes after repeated doublings (Stanulis-Praeger, 1987; Shiels et al., 1999; Lanza et al., 2000).

Replicative senescent cells exhibit an altered behaviour and phenotype as compared to their proliferating counterparts. Senescent fibroblasts possess a larger, flatter morphology (Bowman et al., 1975; Sherwood et al., 1988), with an enlarged nucleus (Mehta et al., 2007; Mitsui and Schneider, 1976), increased adhesion to the extra cellular matrix, fewer cell–cell contacts (Campisi, 2000; Narita et al., 2003; Ben-Porath and Weinberg, 2004) and increased aneuploidy (Benn, 1976; Sherwood et al., 1988; Mukherjee et al., 1995). Moreover, in recent years many studies have demonstrated alterations to the genome organisation of senescent cells (Bridger and Foster, 2021). Exit from the cell cycle into senescence is also accompanied by changes in chromatin modifications (Rai and Adams, 2013) i.e., methylation and acetylation (Wilson and Jones, 1983; Singhal et al., 1987; Imai and Kitano, 1998; Lander et al., 2001; Wagner et al., 2001; So et al., 2006; Dimauro and David, 2009; Grandinetti et al., 2009) with CpG islands being globally demethylated (Cheng et al., 2017) and other specific CpG islands being hypermethylated (Cruickshanks et al., 2013). Core histones are decreased (Lee et al., 2020). Other chromatin remodelling alterations include specific reduction in H3K4me3, H3K9me3, H4K20me3, H3K27me3, H3K36me3, (Di Micco et al., 2011; Salama et al., 2014, Sen et al., 2016; Cheng et al., 2017), deactylation of H4K16 (Contrepois et al., 2012), and H3K56, with increased levels of H3K9ac and H3K27ac associated with specific gene promoters (Gorgoulis et al., 2019; Zhang et al., 2020; Yi and Kim, 2020). With respect to heterochromatin, there is evidence of increased heterochromatisation (Kreiling et al., 2011), with the formation of specific senescence associated heterochromatin foci (SAHF; Chandra and Narita, 2013). But SAHFs are not found in all types senescent cells (Sati et al., 2020). They are a marker of OIS and are created through changes in nuclear pore density at the nuclear edge (Boumendil et al., 2019) and lamin B1 reduction, altering the positioning of genomic regions at the nuclear periphery (Sadaie et al., 2013). SAHFs contain regions of condensed chromatin associated with late replicating, gene-poor regions of the genome. Their function is not clear but they do represent an alteration to genome organisation and regulation (Sati et al., 2020).

In proliferating cells gene-poor regions of the genome are attached to the nuclear envelope through interactions with B-type lamins and other cell specific nuclear envelope proteins (de Las Heras et al., 2017). These specific regions have been termed Lamina Associated Domains (LADs) and have been selected by using an exogenous construct containing the gene for lamin B1 combined with a bacterial enzyme DNA adenine methyltransferase that specifically methylates any associated DNA so that it can be isolated and sequenced (van Steensel and Belmont, 2017). LADs comprise about one third of the genome and so are highly significant in organising the genome within cell nuclei and have some overlap with nucleolar associated domains (NADs; Németh et al., 2010; van Koningsbruggen et al., 2010). NADs remain very similar in replicative senescent embryonic fibroblasts (Dillinger et al., 2017). However, some LADs are released from the nuclear edge with the loss of lamin B receptor (Arai et al., 2019) and lamin B1 in senescence (Shimi et al., 2011; Freund et al., 2012; Hutchison, 2012; Lukášová et al., 2017, 2018). Indeed, chromosome 18 is less attached to the nucleoskeleton than chromosomes 1, 13, 17 in replicative senescent cells, as is *CTNNA1* gene compared to *CNDD1* (cyclin D1) (Godwin et al., 2021). The LADs in OIS cells are different to proliferating and quiescent cells (Lenain et al., 2017). Chromosome conformation capture has also identified global changes in genome organisation in OIS cells (Chandra et al., 2015; Criscione et al., 2016; Zirkel et al., 2018). However, HiC chromosome conformation capture experiments unequivocally demonstrated that OIS is not comparable to RS (Sati et al., 2020). The interactions between topologically associated domains (TADs) A (active) and B (inactive) compartments are different in the two types of senescence, with OIS and RS both having increased long-range interactions of genomic regions, but with RS cells displaying more A to B interactions, indicating decreased genome compaction (Sati et al., 2020).

Since the genome is highly organised within the nuclei of proliferating cells (Croft et al., 1999) it came as no surprise to find non-random genome organisation through whole chromosome positioning analysis in non-proliferating cells, serum starved quiescent and replicative senescent cells. Bridger et al. (2000) demonstrated that the gene-poor chromosome 18, located at the nuclear periphery in proliferating human dermal fibroblast (HDF) skin cells, was found deep within nuclei, attached to the nucleoskeleton in non-proliferating cells. It was not clear if chromosome positioning would be different in senescent cells when compared to a cell’s other pathway to leave the cell cycle, quiescence. However, when opening up the panel of chromosomes studied, it became obvious some chromosomes do not move at all when entering G0, some move to the nuclear periphery and some to the nuclear interior (Bridger et al., 2000; Meaburn et al., 2007; Mehta et al., 2007, 2010; Gillespie et al., 2015; Belak et al., 2020). Many of these chromosomal locations positions have been confirmed by chromosome conformation capture data (Das et al., 2020). This change in location of the chromosomes would place the chromosomes into a different nuclear compartment, exposing them to a alternative nuclear environment and interactive anchorage points. It is strongly supported in the literature that chromosome and gene spatial positioning is a further epigenetic mechanism for regulating gene expression (Sivakumar et al., 2019). Thus understanding how the genome, chromosomes and genes behave in senescent cells will be an important step in revealing the important differences in young proliferating cells and replicative senescent cells.

By using our FISH chromosome mapping assay, we have demonstrated in young proliferating HDFs whole chromosomes move to new locations within cell nuclei when an external stimulus, such as being placed in low serum for 15 min (Mehta et al., 2010). This is rapid relocalisation requires nuclear myosin 1β (NMIβ), presumably within a nuclear motor complex with actin, using energy (Mehta et al., 2008; Bridger, 2011). Interestingly, both chromosomes 18 and 13 move rapidly to the nuclear interior, in similar locations to where they are located in senescent HDF (Bridger et al., 2000; Meaburn et al., 2007). Further, positioning of all human chromosomes in quiescent HDFs determined that the organisation of chromosome territories in interphase nuclei still remains radial as it is in proliferating cells, but the territories of some chromosomes such as 1, 6, 8, 10, 11, 12, 13, 15, 18, and 20 re-localise and alter positions as the cells enter a state of quiescence (Mehta et al., 2010). Chromosomes are also relocated to areas of DNA repair foci via nuclear motors containing nuclear myosin 1β (Mehta et al., 2013; Kulashreshtha et al., 2016).

To assess whether global spatial repositioning of individual whole chromosomes occurs in replicative senescence of HDFs we have further employed the individual whole chromosome positioning assay (Clements et al., 2016) and in combination with previous studies revealed the nuclear positions of all human chromosomes in senescent primary HDFs. Here, we identify that some chromosomes are found in different nuclear locations, compared to proliferating HDF. More interestingly, we have demonstrated that this was not simply due to exiting the cell cycle since there were clear differences in the spatial positioning of chromosome territories between cells made quiescent and cells that have become senescent by serial passage. Most notable of these is chromosome 10. As a further demonstration of the senescence-specific nature of this positioning, no chromosome movement was apparent for chromosome 10 after placing senescent cultures into low serum. This was not surprising since NMIβ, which is known to be involved in whole chromosome movement, had an altered distribution to proliferating cells, forming aggregates.

This study here, in combination with other studies, delineates the positioning patterns of the chromosomes within senescent HDFs and reveals that territories of chromosome 10, sit in opposing locations in senescent HDFs as compared to the same cell line made quiescent by serum starvation. Thus, the positioning of chromosome 10 could be considered a new biomarker to delineate between the two non-proliferating cell statuses i.e., senescence and quiescence, and easy to establish as a robust, but quick assay to differentiate between quiescent and senescent cells.

## Materials and Methods

### Cell Culture

Human dermal fibroblasts (2DD, Bridger et al., 1993 and 1HD, Bridger et al., 1998) were grown in Dulbecco’s Modified Eagles Medium supplemented with 10% newborn calf serum (NCS). The cells were passaged twice weekly so that they never became contact inhibited and were used at a high passage number >30, where the majority (95%+) of cells were negative for the proliferation marker pKi-67 (Kill et al., 1994). To make cells quiescent HDF were placed in 10% NCS for 48 h and this was washed out and the cells placed in 0.5% NCS for 7 days. For cultures to be deemed proliferating >65% of the cells had to be Ki67+ and were never passaged beyond passage 15. Quiescent cultures were generated by treating cells with 0.5% NCS/DMEM for 7 days. In order to subject the cells to a heat-shock they were incubated at 42°C for 1 h.

### Two-Dimensional Fluorescence *in situ* Hybridisation

Harvested HDFs were initially allowed to swell in 0.075M KCl and then fixed in ice-cold 3:1 (v/v). methanol:acetic acid. The suspension was placed onto glass microscope slides and aged for two days at room temperature. The fixed cells were dehydrated by subjecting them to an ethanol series (100, 90, and 70%, 5 min each). For denaturing, the slides were placed in 70% formamide, 2X SSC, pH 7.0, at 70°C for 2 min. After denaturation, the slides were immediately plunged in ice-cold 70% ethanol for 5 min and then taken through the ethanol series and air-dried.

Directly labelled total human chromosome DNA probes (Appligene Oncor) were denatured by incubating at 70°C for 10 min followed by 30 min reannealing at 37°C. Hybridisation of probe to sample took place over 18 h in a humidified chamber. The slides were washed three times for 5 min each in 50% formamide, 2X SSC, pH 7.0 at 45°C and then with 0.1X SSC prewarmed at 60°C.

### Three-Dimensional Fluorescence *in situ* Hybridisation

For 3D-FISH, cells were grown for 2 days on sterile glass “SuperfrostTM” slides at 37°C, 5% CO2 at a starting density of 1 × 10^5^ cells/slide. Then washed in 1× PBS and fixed in 4% paraformaldehyde (w/v). Cells were permeabilised with 0.5% Triton-X100 (v/v) and 0.5% saponin (w/v) in 1X PBS solution for 20 min at room temperature and then rinsed. The slides were incubated then in a solution of 20% glycerol for at least 30 min at room temperature prior to being snap-frozen in liquid nitrogen for 15–30 s before being stored at −80°C. Chromosome painting probes were denatured at 75°C for 10 min and then allowed to re-anneal at 37°C for 10 min. The freeze–thaw process in liquid nitrogen, as described before was repeated for further 4–5 times with soaking the slides in 20% glycerol between each freeze–thaw. Excess glycerol was washed from the slides using three changes of 1X PBS for 10 min each, followed by depurination in 0.1N HCl for 5 min at room temperature. Excess acid was washed away with 2X SSC for 15 min with three changes of the buffer and then slides were incubated in 50% formamide, 2X SSC, pH 7.0 solution overnight. The slides were denatured by incubation in denaturation buffer A (70% formamide, 2X SSC, pH 7.0) pre-warmed at 73°C for precisely 3 min. The slides were then rapidly transferred to denaturation buffer B (50% formamide, 2X SSC, pH7.0) pre-warmed at 73°C for 1 min. The slide was immediately presented to the probe on the coverslip and incubated in a humidified chamber at 37°C for 2 days and washed as for 2D-FISH.

### Indirect Immunofluorescence

The FISH slides were incubated with anti-Ki-67 antibody (1:1500 dilution, Novacastra) for 1 h at 37°C. After washing in phosphate buffered saline, the slides were incubated in the swine anti-rabbit-TRITC secondary antibody (1:30 dilution, DAKO) for 1 h at 37°C. After washing in 4X SSC, the slides were mounted and counterstained with 4, 6-diamidino-2-phenylindole (DAPI) in Vectashield mounting medium (Vecta Laboratories).

For nuclear myosin 1β staining, cells were grown on 13 mm glass coverslips and fixed in ice-cold methanol:acetone (1:1) on ice for 10 min. Dual staining experiments were performed with mouse anti-pKi-67 and rabbit anti-nuclear myosin 1β (Sigma), diluted to 1:1,500 and 1:50 in PBS/1%NCS (v/v), respectively for 1 h at room temperature. After washing, secondary antibodies were employed: Swine anti-rabbit conjugated to TRITC (DAKO) and donkey anti-mouse (Jackson’s laboratories) conjugated to FITC were diluted 1:30 and 1:70 in PBS/1%NCS (v/v), respectively and left for 1 h at room temperature in the dark. Slides were mounted and counterstained in Vectashield containing DAPI.

### Microscopy and Image Capture

After 2D FISH, interphase nuclei were examined and imaged using a Leica fluorescent microscope with a 100× oil immersion lens (Leica). Random pKi-67 negative nuclei were imaged. Grey-scale images of these nuclei were captured from the microscope using Photometrics cooled charged-coupled device (CCD) camera. These images were pseudocoloured and merged using Digital Scientific software, the Quips Pathvysion, Smart Capture VP V1.4

The images of 3D nuclei, prepared by 3D FISH, were captured using a Nikon confocal laser scanning microscope (TE2000-S) equipped with a 60X/1.49 Nikon Apo oil immersion objective. The microscope was controlled by Nikon confocal microscope C1 (EZ – C1) software version 3.00. Stacks of optical sections with an axial distance of 0.2 μm were collected from random nuclei. Stacks of 8-bit grey-scale 2D images were obtained with a pixel dwell of 4.56 and 8 averages were taken for each optical image.

### Image Analysis

#### 2D-FISH

Fifty nuclei for each chromosome were analysed using a bespoke erosion analysis script in IPLab as described in Croft et al. (1999), a gift from Prof Wendy Bickmore, MRC Human Genetics Unit. The script divides nuclei into five shells of equal area and measures the pixel intensity of the DAPI signal and the chromosome probe in each of the five shells. The probe signal is normalised by dividing the percentage of the probe signal by the percentage of DAPI signal in each shell. Histograms were plotted and standard error bars representing ± standard error of the mean (SEM) are shown. Statistical analyses were performed using the two tailed Student’s *t*-tests. The necessary controls have been performed whereby a new researcher will repeat chromosomes already delineate to be sure that the results are reproducible and consistent between the data sets.

#### 3D-FISH

The positioning of chromosomes in relation to the nuclear periphery was assessed by measurements obtained using Imaris Software (Bitplane scientific solutions), whereby the distance between the geometric centre of each chromosome territory and the nearest nuclear edge was measured. Measurements for at least 20 nuclei were performed for each chromosome. Frequency distribution curves were plotted with the distance between the centre of chromosome territory and the nearest nuclear periphery on the *x*-axis and the frequency on the *y*-axis. Statistical analyses were performed using the two tailed Student’s *t*-tests.

### RNA Extraction

RNA was extracted from samples using the MP24 fastprep (MP Biomedical) system, following the manufactures protocol. superRNAsin (Ambion) was added to each sample prior to snap freezing and storage at −80°C. *N* = 4 biological replicates for each of proliferative, quiescent and replicative senescent RNA samples was analysed to monitor changes in transcript abundance. Paired immuno-fluorescence analysis of cells using Ki-67 was performed to determine the status of each culture.

### Microarrays

Microarray analysis was carried out using Op Human ReadyArray HS1200 slides (Microarrays Inc.), with the 3DNA Array 900 labelling kit (Genisphere). One microgram of RNA derived from proliferative, quiescent, or replicative senescent 2DD cultures was resuspended in 5 μl H_2_O and 1 μl of RT primer was added with the correct dendrimer target sequence for labelling of the samples on the array. The mixture was heated to 80°C for 5 min to denature, placed on ice for 2 min, and the following reagents were added to each reaction: 2 μl of first-strand buffer, 1 μl of 0.1 M DTT, 0.5 μl of SUPERase-In (provided with the 3DNA 900 kit), 0.5 μl of dNTP mix (provided with the 3DNA 900 kit), and 0.5 μl of SuperScript III (Invitrogen). The reaction was incubated for 2 h at 42°C and stopped by adding 1 μl of 1 M NaOH/100 mM EDTA and incubating at 65°C for 10 min to denature the cDNA/RNA hybrids and degrade the template RNA. The reverse transcription reaction was then neutralised by adding 1.2 μl of 2 M Tris-HCl pH 7.5. 1 μl of H_2_O was then added to each cDNA sample, the samples were mixed, and then 1 μl of sample was then assessed using the Qubit^®^ single-stranded DNA assay on a Qubit^®^ 1.0 Fluorometer, to check that a sufficient quantity of cDNA was present.

Samples were then mixed to form the hybridisation mix for the microarray slides. About 12.7 μl of each cDNA were mixed with 40 μl of 2× SDS-based hybridisation buffer and 14.6 μl of H_2_O, to a final volume of 80 μl. The mixture was heated to 80°C for 10 min in order to denature secondary structures, and then it was cooled to 60°C in preparation for addition to the slide.

The array slides were pre-hybridised at 65°C for 20 min with 3.5 × SSC, 0.1% SDS and 10 mg/ml BSA solution in a volume of 50 ml. The slides were washed in MilliQ water for 1 min, in isopropanol for 1 min, and dried using a Microarray High Speed centrifuge (Arrayit Corporation). The slide was then pre-scanned for the second time with the GenPix 5.1 scanner to check it was clean and undamaged, before the hybridisation was set up. The microarray slide was then placed into a clean SlideBooster (Advalytix) on a layer of 45 μl AS100 AdvaSon coupling solution (Beckman Coulter), with 60 μl more in the thumb hole at the base of the slide. The wells of the slide booster were each filled with 500 μl of AdvaHum AM102 humidifying solution (Beckman Coulter), and a 24 mm × 60 mm LifterSlip was placed on top of the microarray slide. The assembly was then pre-warmed to 55°C, and when it reached temperature, the hybridisation solution was pipetted underneath the LifterSlip. The microarrays were then hybridised for 16 h. The slides were then washed in 2 × SSC, 0.2% SDS at 55°C for 10 min, followed by a wash in 2 × SSC at room temperature for 10 min, followed by a wash in 0.2 × SSC at room temperature with orbital rotation of 150 rpm for 10 min. The slides were then dried using a Microarray High Speed centrifuge (Arrayit Corporation). For each slide, 2.5 μl of the Cy3 capture reagent was mixed with 2.5 μl of the Cy5 capture reagent, with 40 μl of 2 × SDS-based hybridisation buffer and 35 μl of H_2_O, to a final volume of 80 μl, to make the second hybridisation mix. This was heated at 80°C for 10 min, and then cooled to 55°C in preparation for addition to the slide. The SlideBooster was assembled as before, and pre-warmed to 50°C. When it was warm, the second hybrisation mix was added, and the arrays were incubated for 4 h. Array slides were washed in 2 × SSC, 0.2% SDS at 55°C for 10 min, followed by a wash in 2 × SSC at room temperature for 10 min, followed by a wash in 0.2 × SSC at room temperature for 10 min. The slides were then dried using a Microarray High Speed centrifuge (Arrayit Corporation).

### RNA-Seq

Two RNA-seq replicates were used for each sample type, as recommended by the ENCODE Consortium’s Standards, Guidelines and Best Practices for RNA-Seq.^1^ RNA was isolated using the FastPrep-24 instrument (MP Biomedicals) according to the manufacturer’s instructions. RNA integrity was determined using the Bioanalyzer (Agilent Technologies) with RNA having an RNA integrity number above 9.0 used for further analysis. For sequencing library synthesis, polyadenylated RNAs were purified using oligo dT-beads (Invitrogen) with random hexamers, and used as primers for the cDNA library construction prior to paired-end sequencing. Sequencing was performed using Illumina GxII platform. All sequencing reactions resulted in the generation of 50 bp paired-end reads. RNA-seq reads were subjected to quality control using the standard Illumina pipeline. Raw sequence reads were mapped against the GRCh37 assembly reference genome using the following command to TOPHAT 2. No trimming of reads was performed prior to mapping. The BAM files produced by TOPHAT 2 were then imported into SEQMONK.^2^ The feature probe generator function in SEQMONK was used to generate probes based on mRNA annotations from ENSEMBL. The number of reads that mapped to each probe was then quantitated, and normalised using the widely used RPKM method. A constant value of 0.05 was added to each value in order to prevent cases of division by zero when calculating FC values. To note gold standard senescent biomarker genes p16^*INK*4*a*^ and p21^*CIP*1^ are both upregulated in the senescent cells used, as well 39 other genes associated with senescence CellAge: The Database of Cell Senescence Genes.^3^

## Results

### Positions of Human Chromosomes in Senescent Human Dermal Fibroblasts

**FIGURE 1:**
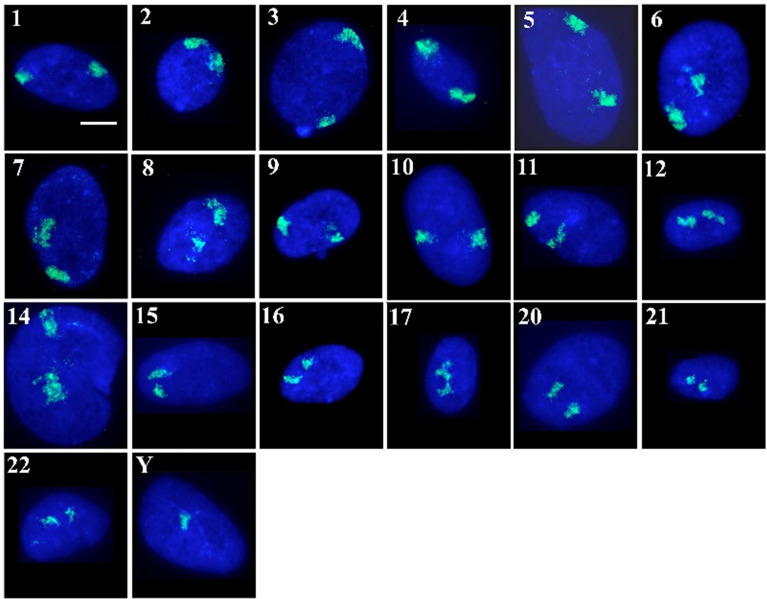
Human chromosome territories in normal senescent human dermal fibroblast nuclei: Representative images displaying the spatial arrangement of human chromosome territories (in green) in senescent interphase nuclei of fibroblasts, stained with DAPI (blue). The numbers/letters by the side of each nucleus indicates the chromosome hybridised to by fluorescence *in situ* hybridisation. All the cells were grown in 10% new born calf serum and were found to be negative for the proliferation marker pKi-67. Scale bar = 10 μM.

In this study, we now complete the radial mapping of all human chromosomes in RS HDF nuclei using whole chromosome painting probes and FISH, followed by analysis of interphase position through a bespoke erosion script for 2D positioning and 3D analysis of the reconstruction of optical sections. To achieve this HDFs were harvested and fixed for standard 2D-FISH and hybridised with whole chromosome painting probes for chromosomes 1–12, 14–17, 20–22, and Y. The positions of the other chromosomes has been completed previously in other studies (see Supplementary Table 1). Senescent cells within late passage primary cultures, which had been grown in 10% serum and were not permitted to reach confluency at any stage of their passaging, were identified by the lack of the proliferative marker pKi-67 (Clements et al., 2016; Figure 1).

**FIGURE 2:**
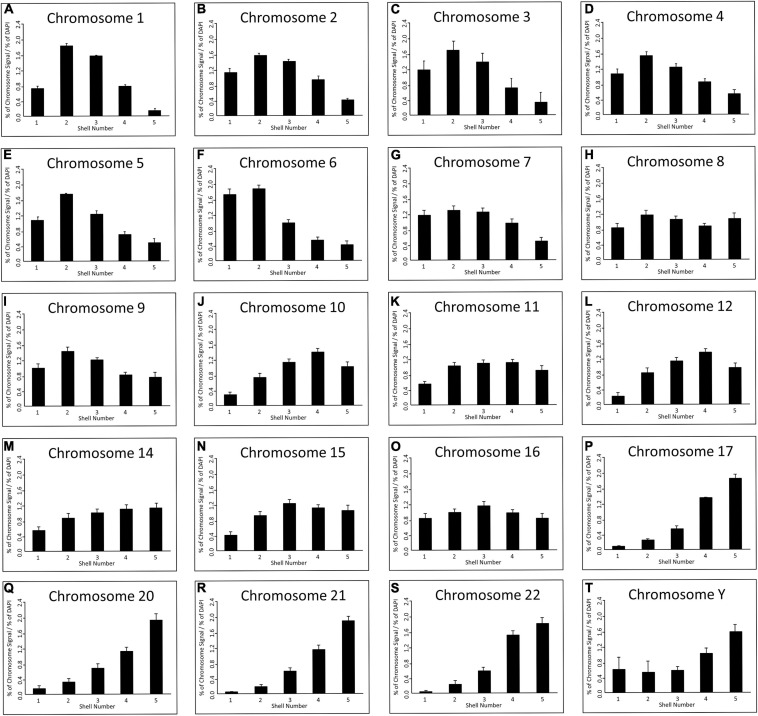
Spatial distribution of human chromosome territories in normal senescent fibroblast nuclei: Digital images (>50 nuclei) for each chromosome were analysed by a simple erosion analysis script (Croft et al., 1999; Clements et al., 2016). The script divides the cell nuclei into five shells of equal area and measures the % of signal intensity from both chromosome signal and the DNA (DAPI). The % of chromosome signal is normalised by division of the % of DAPI in each of the eroded shells (*y*-axis); and the shell numbers of 1–5 are on the *x*-axis. The error bars represent the standard error of mean (SEM). Shells 1 and 2 denote the nuclear periphery and shells 4 and 5 the nuclear interior. chromosomes as indicated above each graph in senescent nuclei as visualised by FISH and specific probes and erosion analysis. **(A)** Chromosome 1, **(B)** Chromosome 2, **(C)** Chromosome 3, **(D)** Chromosome 4, **(E)** Chromosome 5, **(F)** Chromosome 6, **(G)** Chromosome 7, **(H)** Chromosome 8, **(I)** Chromosome 9, **(J)** Chromosome 10, **(K)** Chromosome 11, **(L)** Chromosome 12, **(M)** Chromosome 14, **(N)** Chromosome 15, **(O)** Chromosome 16, **(P)** Chromosome 17, **(Q)** Chromosome 20, **(R)** Chromosome 21, **(S)** Chromosome 22, **(T)** Chromosome Y.

As has been executed previously for HDF (Croft et al., 1999; Bridger et al., 2000; Boyle et al., 2001; Meaburn et al., 2005, 2007; Mehta et al., 2010; Bikkul et al., 2018), in order to position the chromosome territories images of 50+ random nuclei were captured (Figure 1) and individual chromosome positions assessed by using an erosion analysis script which measures the intensity of fluorescent signal in five concentric shells of equal area, made by eroding the nuclear outline from the edge to the nuclear interior. The position of whole chromosomes is revealed by normalisation through dividing the signal of the chromosome within a shell with the measured signal for the amount of DNA stained by DAPI (Croft et al., 1999; Clements et al., 2016), and the data plotted as histograms (Figure 2). The shape of the graph indicates where the chromosomes are located and so we assign a category to each shape of graph. With a skew towards shells 1 and 2 the chromosome is said to be peripheral, with a skew towards shells 4 and 5 the chromosome is said to be interior and where the histogram peaks in shell 3 the chromosome is said to be intermediate in nuclear location (Figure 2 and Supplementary Table 1).

Since this experiment completes the mapping of all the chromosomes by 2D-FISH in young proliferating HDF (Croft et al., 1999; Meaburn et al., 2007; Mehta et al., 2010), quiescent (Bridger et al., 2000; Mehta et al., 2010) and senescent HDF (Bridger et al., 2000; Meaburn et al., 2007; this study); we are now in a position to note changes in chromosome location between these cell cycle statuses. This reveals that there are major nuclear location changes for whole chromosome territories in senescent cells, as compared to previously published data for young proliferating HDF using the same cells and methods. The categorisation of all the chromosome positions in HDFs in Supplementary Table 1 has been collated from a number of papers, from two different laboratories but, importantly, using the same methodology and analysis script. Furthermore, many of the chromosome positions are confirmed using 3D-FISH, confocal imaging and 3D measurement and analyses. The position of many chromosomes are unaffected by entrance in to senescence. However, chromosomes 1, 5, 6, 10, 12, 15, and 16 occupy differential locations in senescent cells to that of young proliferating HDF cells (Supplementary Table 1). These are in addition to chromosome 18 and 13 that have been shown previously to be relocated in senescent HDFs (Bridger et al., 2000; Meaburn et al., 2007; Supplementary Table 1). Chromosomes 1, 5 and 6 relocate from an intermediate location to a peripheral location in senescent cells, whereas 12, 13, and 18 move from a peripheral location to the nuclear interior. Chromosome 16 relocates from an interior location to an intermediate location and chromosome 10 relocates from an intermediate location to an interior location (Supplementary Table 1).

Since we know that chromosomes reorganise when cells exit the cell cycle in quiescence (Bridger et al., 2000; Mehta et al., 2010), we also wanted to determine if the repositioning events we detect are common to cells that have just exited the cell cycle or are specific to senescence. When the comparison between positioning categories was made between young HDF made quiescent by 7 days serum-starvation and the senescent cells, there are also some differences, with chromosomes 5, 8, 10, 11, 15, 16, and 20 having chromosomes in different spatial categories (Supplementary Table 1), e.g., for chromosome 5 the territories are in an intermediate location in quiescent HDF but towards the nuclear periphery in senescent cells. However, there are two chromosomes, chromosomes 10 and 15, which are located in different nuclear compartments in proliferating, quiescent and senescent cells (Supplementary Table 1). These repositioning events do not represent a general reorganisation of the genome since there are also chromosomes that do not change their location category at all; the peripheral chromosomes are 2, 3, 4, 7, 9, and X and the interior chromosomes are 14, 17, 19, 21, 22, and Y.

**FIGURE 3:**
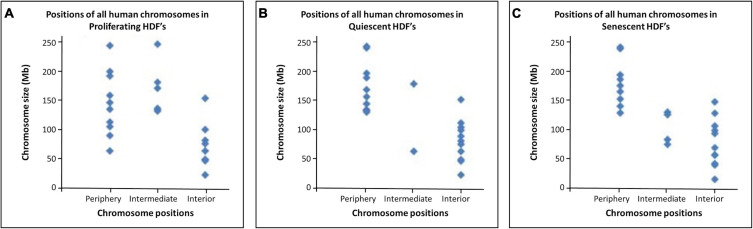
Relationship between chromosome size and nuclear location within proliferating, quiescent and senescent HDFs: The size (Mb) of each chromosome falling within a positioning category (nuclear periphery, at an intermediate location, or in the nuclear interior) in proliferating, quiescent and senescent cells are displayed in panels **(A–C)**, respectively.

When the categorised chromosome positions are plotted against chromosome size in Mb (Figure 3) it is very noticeable that in quiescent and senescent cells the distribution of chromosome territories adheres much more to a size-distribution than in proliferating cells. Thus, it appears in non-proliferating cells chromosome territories are positioned more according to their size with larger chromosomes at the nuclear periphery and smaller chromosomes within the nuclear interior.

### Chromosome 10 Occupies Differential Locations in Young Proliferating, Young Quiescent and Old Senescent Human Dermal Fibroblasts

**FIGURE 4:**
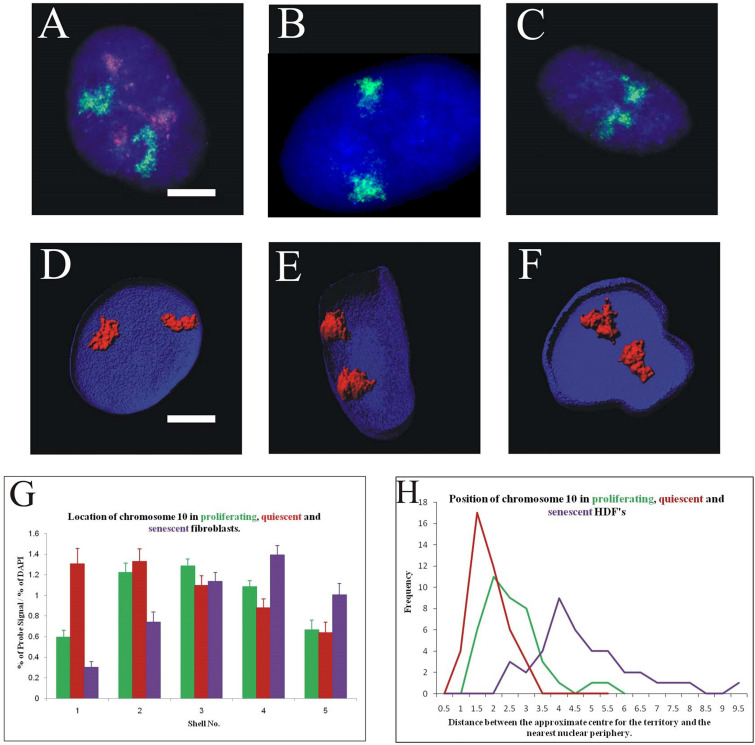
Differential location of chromosome 10 territories in proliferating, quiescent and senescent cell nuclei: Panels **(A–C)** represent cell nuclei that have been subjected to 2D-FISH, displaying chromosome territories (green) and the proliferation marker pKi-67 (red). Panels **(D–F)** display 3D reconstructions of cell nuclei that have been prepared for 3D-FISH and optical imaged using a confocal laser scanning microscope. Chromosome territories are in red the nuclei delineated in blue (DAPI). Scale bar = 10 μm. Panel **(G)** displays comparative histograms of the position of chromosome 10 in proliferating (green), quiescent (red), and senescent (purple) nuclei, as determined by 2D FISH and erosion script analysis. Error bars represent standard error of the mean (SEM). Panel **(H)** displays comparative frequency distributions of measurements for the position of chromosome 10 in proliferating (green), quiescent (red), and senescent (purple) in 3D preserved nuclei. Measurements have been made from the geometric centre of each chromosome territory to the nearest edge in 3D. Unpaired, unequal variance, two-tailed Student’s *t*-test at 95% confidence interval (*p* < 0.05) has been performed.

The most interesting chromosome with respect to thedifference between the non-proliferative states was chromosome 10; which occupies an intermediate position in young proliferating cells (Figures 4A,D), but a peripheral location in when placed in low serum for 15 minutes - 7 days (Figures 4B,E) and localises at the nuclear interior in senescent cells (Figures 4C,F). These positions were confirmed both in 2D (Figure 4G) and 3D analyses (Figure 4H). The normalised percentage chromosome signal is greatest in shells 1 and 2 for quiescent HDF, in shells 2 and 3 for proliferating HDF and in shells 3 and 4 for senescent cells. In 3D analyses, optical sections of 20 nuclei were collected on the confocal microscope and reconstructed using Imaris software. The geometric centre of the chromosome territory in 3D was determined and a measurement made to the nearest nuclear periphery, as delineated by DAPI staining. The measurements were binned in 0.5 μm increments and a frequency distribution created for the measurements of chromosome 10 in young proliferating, young quiescent and late passage senescent HDF. In the frequency distribution, the peak for chromosome 10 in quiescence cells is the closest to the nuclear periphery, followed by proliferating cells and then with the peak for the senescence measurements being the furthest away from the nuclear periphery.

This large difference in the nuclear localisation of chromosome 10 provides a novel and robust new biomarker for differentiating between quiescent and senescent cells.

### Differences in Expression of Genes located on Chromosome 10 in Proliferating, Quiescent and Senescent HDFs

**FIGURE 5:**
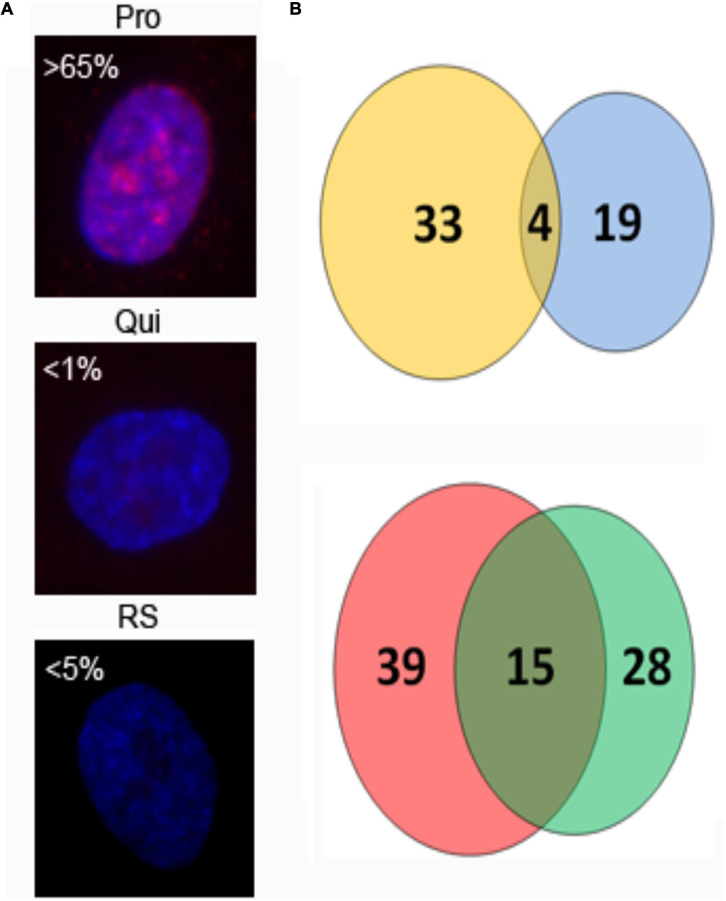
Number of chromosome 10-associated genes with changed transcript abundance in quiescent and replicative senescent fibroblasts. 2DD fibroblast cultures were immuno-labelled for the proliferative marker Ki67 [panel **(A)**, red] to show growth status. Chromatin is counterstained in DAPI (blue). Percent Ki67+ cells are indicated and show the presence of Ki67 in proliferating (Pro), quiescent (Qui), and replicative senescent (RS) cultures. RNA was extracted from parallel cultures and used in microarray analysis to identify genes that had increased **(B, top panel)** or decreased **(B, lower panel)** transcript abundance from chromosome 10 as 2DD cells become quiescent or replicative senescent. Thirty three genes increased transcript levels in replicative senescent samples (yellow circle) and 19 in quiescent samples (blue circle) with 4 genes in common between the data sets. Thirty nine genes exhibited significantly decreased transcript levels in replicative senescent samples (red circle), 28 in quiescent samples (green circle) with 15 of these common between the data sets.

The differential locations of chromosome 10 territories in non-proliferating cells provide an excellent model system in which to study more detailed aspects of chromosome behaviour and the importance of spatial positioning to regulate function. Using this model, we extracted total RNA from proliferating, quiescent and replicative senescent fibroblasts to determine what effect the relocalisation of chromosome 10 has on transcript abundance from this chromosome using a microarray analysis. Our data demonstrate that 33 genes increase transcripts and 39 genes have a significant decrease in transcripts when senescent cells are compared to proliferative cells (Figure 5). Interestingly, only four genes with increased transcript levels were found in both quiescence and senescent cells, whereas 15 genes decreased transcript levels in both compared to proliferative cells (Figure 5). This demonstrates that the repositioning of the chromosome 10 into the nuclear interior senescence does not mean that genes will be repressed. Although only 72 genes were identified to have significantly changed transcript abundance from chromosome 10 during senescence we were able to identify specific pathways that were enriched for. We identified that there are changes in transcript abundance related to cell cycle control and steroid hormone biosynthesis, however these pathways were also enriched for in quiescent cells as well, further suggesting that the change in chromosome location was unrelated to gene expression changes. This is supported by RNA-seq data that also show up and down-regulation of genes on chromosome 10 when it is either at the nuclear periphery or interior in non-proliferating cells (Supplementary Table 1).

### Chromosomes in Senescent Cells Cannot Be Induced to Relocate After a Stimulus

**FIGURE 6:**
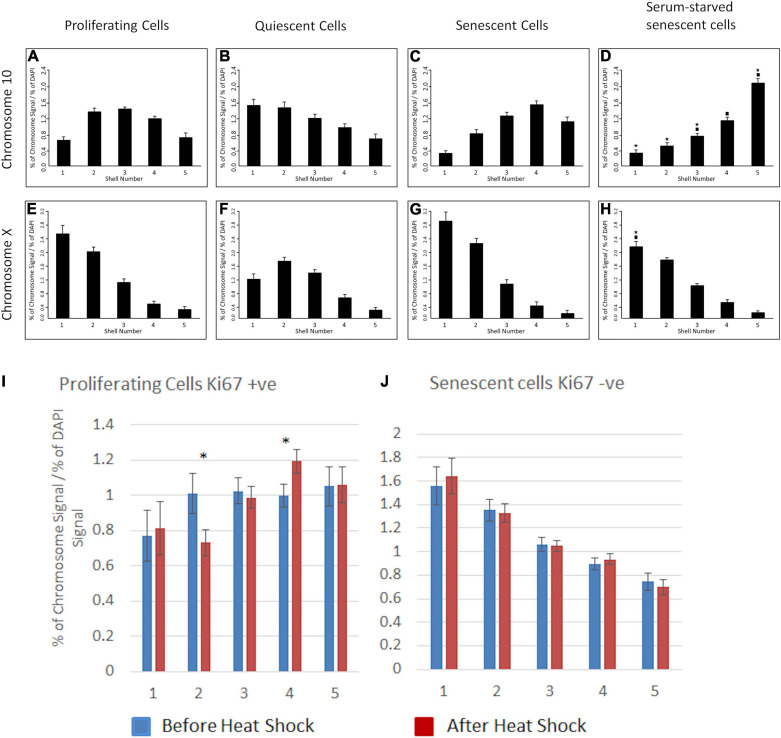
Chromosomes 10, X or 11 do not relocate to new nuclear locations in senescent nuclei upon serum removal or heat shock. For senescent cells HDFs were grown in 10% NCS until the culture became mostly comprised of non-confluent senescent cells as determined by the absence of anti-pKi-67. Young proliferating cells were collected from early passage cultures where anti-Ki67 staining was in over 65% of cells. The cultures were serum-starved by incubation with 0.5% NCS for 7 days (chromosome 10 and X) or subjected to a heat-shock (42°C, 1 h, chromosome 11). Positions of chromosomes 10 and X were determined using 2D-FISH erosion analysis and anti-Ki67 staining to differentiate between proliferating and senescent cells. Chromosome 10 **(A–D)**, chromosome X **(E–H)**, chromosome 11 **(I,J)**. Panels **(A–H)**: The asterisks indicate statistical difference (*p* < 0.05), as assessed by Student’s *t*-test, to the normal quiescent cells. The filled-in squares indicate statistical difference (*p* < 0.05) to normal senescent cells grown in 10% serum. In panels **(I,J)** asterisks indicate statistical difference (*p* < 0.05), as assessed by Student’s *t*-test for before and after heat-shock.

We have demonstrated that specific chromosomes can be actively repositioned rapidly upon a stimulus in young proliferating cells via nuclear motors comprising nuclear myosin 1β (Mehta et al., 2010). We sought to investigate whether chromosomes can be induced to actively relocate in cells that have become senescent. Thus, we placed late passage cultures into low serum to induce chromosome repositioning. We analysed the nuclear positions of both chromosomes 10 and X using a standard 2D-FISH assay (Figures 6A–H). We found that in senescent HDFs chromosome 10 territories did not relocate to the nuclear periphery (Figure 6C), where they are found in young quiescent HDF (Figure 6B) but remained within the nuclear interior (Figure 6C). When compared to senescent HDF grown in 10% serum in the senescent cells placed in low serum there was a significant shift ever more towards the nuclear interior (Figure 6D). As expected, the X chromosome territories did not change their position at the nuclear periphery (Figures 6E–H). To investigate a further stimulus we subjected senescent HDFs to a 42°C heat-shock for 1 h with continuous 5% CO_2_, fixed cells for 2D-FISH and analysed the nuclear location of chromosome 11, the chromosome containing a number of heat shock genes. In young proliferating cells (positive for Ki67) significantly relocate chromosome 11 to a new nuclear location, more towards the nuclear interior than the intermediate location in cells after heat shock (Figure 6I). However, there is no movement of chromosome 11 at all in senescent cells when responding to heat-shock (Figure 6J), this correlates with heat shock gene transcription failing in senescent cells (Sabath et al., 2020).

### Differential Nuclear Myosin Iβ Distribution in Normal Proliferating, Quiescent, Senescent HDFs

**FIGURE 7:**
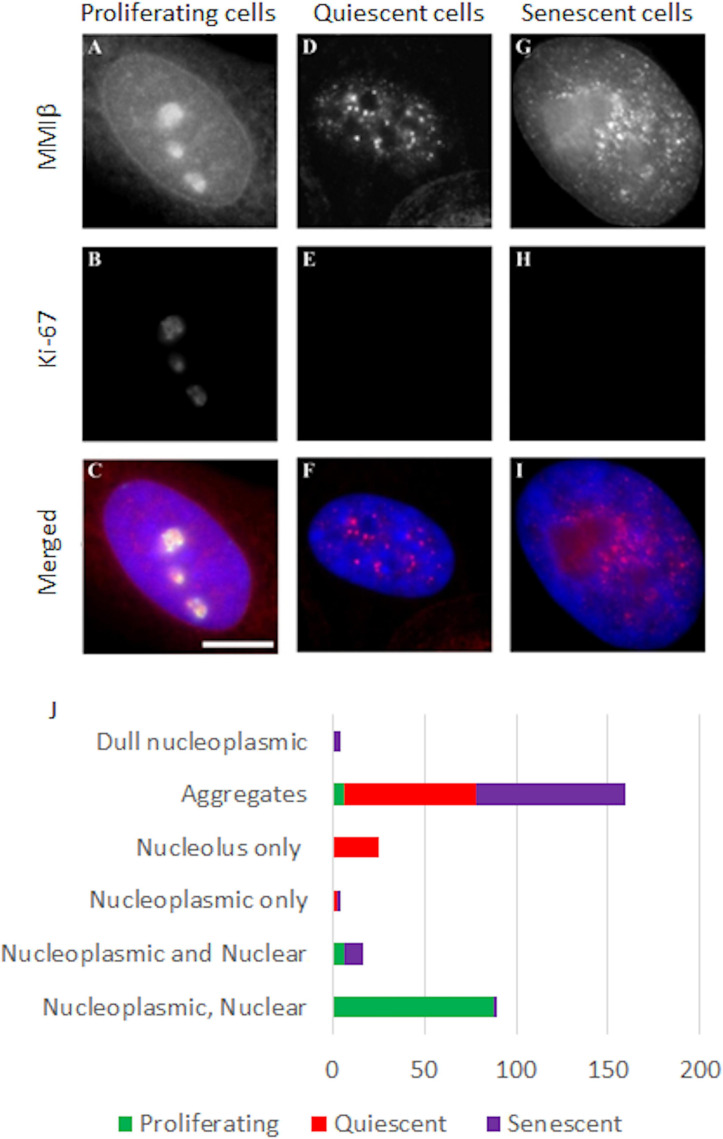
Representative images displaying the distribution of NMIβ in proliferating, quiescent and senescent HDFs. Young, serum starved young and senescent HDF were grown on glass coverslips, fixed with methanol:acetone and co-stained for nuclear myosin 1β [red, **(A,D,G)**] and pKi-67 [green, **(B,E,H)**]. DNA is delineated by DAPI and merged images are displayed in panels **(C,F,I)**. Scale bar = 5μm. **(J)** displays the fractions of the different patterns of nuclear myosin 1β -nucleoplasmic, nuclear rim and nucleolar; nucleoplasmic and nuclear rim, nucleoplasmic only, nucleolus only, aggregates and dull nucleoplasmic. Error with respect to standard deviation can be found in Supplementary Table 2.

We have previously demonstrated that NM1β is required for whole chromosome movement when HDF are placed in low serum (Mehta et al., 2010) and have shown its distribution is considerably altered in quiescent (Mehta et al., 2010) and in Hutchinson-Gilford Progeria Syndrome (HGPS) HDFs (Mehta et al., 2011). Therefore, we questioned if the distribution of NM1β was also affected in senescent HDFs, which could explain the lack of chromosome repositioning in senescent cells, post-stimulus. In proliferating HDFs, NMIβ is found distributed throughout the nucleoplasm, along the nuclear envelope and within the nucleoli (Mehta et al., 2010; Figures 7A–C). When HDFs enter quiescence this distribution of NMIβ is lost and NM1β becomes accumulated in large aggregates throughout the nucleoplasm (Figures 7D–F). In the senescent HDFs, the distribution of NM1β was not as it is in proliferating HDF but was more similar to quiescent cells with large aggregates and some nucleoplasmic stain (Figures 7G–I). More specifically, NMIβ positive cells were analysed for the distribution pattern of NMIβ, and we classified the different distribution patterns (Figure 7J). The fraction of cells in each category was scored in over 200–500 cells in three independent experiments and correlated with the presence of pKi-67 in passage 11 (young passage) cells, or with the absence of pKi-67 in passage 43 (late passage) cells, and in serum starved passage 11 cells (quiescence) (Figure 7J and Supplementary Table 2). Proliferating HDF displayed 87% with a proliferating distribution of NMIβ of a nucleoplasmic, nuclear rim and nucleolar distribution, whereas this dropped to 0.3% and 2.5% in quiescent and senescent cells respectively (Supplementary Table 2). The largest fraction (72%) of NM1β pattern in the quiescent cells was the aggregates-only pattern with 25% displaying NM1β only at the nucleolus. Interestingly, the late passage cultures had 81% of their Ki67 negative cells displaying aggregates of NM1β. Thus, the lack of relocation of chromosomes in response to stimuli in senescent cells is correlated with an altered distribution of NM1β into aggregates.

## Discussion

Using the Bickmore and Perry analysis method of localising chromosome territories in 2D fixed and flattened nuclei, and employing the original analysis script to radially position normalised chromosome signal (Croft et al., 1999; Clements et al., 2016), all human chromosomes in young proliferating HDF (Croft et al., 1999; Boyle et al., 2001; Meaburn et al., 2007) and quiescent HDF (Bridger et al., 2000; Mehta et al., 2010) have been mapped. Many of these chromosomal locations have been confirmed by 3D-FISH and analysis of confocal laser scanning microscopy optical images. The study presented here completes the nuclear positioning of all human chromosomes in normal replicative senescent primary HDF using the same analysis methods. Comparisons of the distribution of chromosomes in replicative senescent nuclei is similar to quiescent nuclei in that there is a definite influence of chromosome size in positioning, with smaller chromosomes towards the nuclear interior and larger chromosomes towards the nuclear periphery (Supplementary Table 1; Sun et al., 2000; Cremer et al., 2001, Bolzer et al., 2005). However, we reveal that chromosome positioning is not entirely equivalent in quiescent and replicative senescent nuclei and that there are specific differences between the two non-proliferating statuses. Most notably, there is one chromosome that is found in opposing nuclear locations in these two types of non-proliferating HDFs. This is human chromosome 10, whose territories are located at the nuclear periphery in quiescent cells and in the nuclear interior in replicative senescent cells. This disparate positioning must be regulated and our hypothesis is that the plethora of genes concerned with proliferation on chromosome 10 (see Deloukas et al., 2004) would need to be regulated differently in the two arrested situations, since one situation is irreversible and the other reversible, with caveats. However, we have demonstrated here that there is expression from chromosome 10 in both quiescent and replicative senescent cells (Supplementary Table 1) – these can be the same genes or different genes. It is no surprise that downregulation of proliferation genes in both non-proliferating situations has occurred but it would be interesting to analyse the method of silencing for genes such as *CDK1* and *SIRT1* on chromosome 10. The reorganisation of chromosome 10 as well as other chromosome such as 18 and 13, may represent the gain or loss of specific long-range chromatin interactions that influence whether fibroblasts proliferate, quiesce or become senescent. Indeed there are large areas of heterochromatin surrounding nucleoli with which association may elicit a silencing effect on chromatin. Therefore, specific genes on chromosome 10 may become irreversibly silenced in senescent cells by relocation to more internal positions. When analysing specific gene expression from chromosome 10 we found some genes become down-regulated in senescent cells that are up-regulated in quiescent HDF (Figure 5 and Supplementary Table 1), further indicating the nuclear edge is not exclusively an area of down-regulation. Interestingly, cells made senescence through stress i.e., stress induced premature senescence (SIPS) seem to display chromosome 10 at the nuclear periphery, where it is located in quiescent HDFs (data not shown). This is similar to the nuclear position of chromosome 10 revealed by analysis of HiC data for OIS in WI38-hTERT fibroblasts (Das et al., 2020). This suggests that different types of senescence may have different positioning patterns for specific chromosomes. Understanding these differences may give us a greater insight into the mechanisms that control genome positioning patterns and health of the cells.

We have shown that movements of whole chromosomes require NMIβ to be present in the correct distribution (Mehta et al., 2010; Bridger and Mehta, 2011; Mehta et al., 2011). Here we show that senescent cells do not have the ability to relocate chromosome 10 to the nuclear periphery upon serum removal nor chromosome 11 towards the nuclear interior upon a heat-shock. We have demonstrated that both non-random movements occur in young proliferating HDFs (Mehta et al., 2010; Figure 6). This strongly implies that the chromosome movement mechanism may not be functional in senescent cells and this finding correlates with the senescent nuclei containing aggregated NMIβ, rather than dispersed NM1β throughout the nucleoplasm, as it is in proliferating cells. Furthermore, our RNA-seq studies also reveal the gene *MYO1C*, encoding NM1β, to be down-regulated in the senescent HDFs (Supplementary Figure 1). Contrary to this, *MYO1C* is not found as a gene upregulated nor associated with senescence in the databases genAGE,^4^ HCSGD (Dong et al., 2017), CellAge^5^ and on the reverse does not come up as a gene that could be used as a reference gene in qPCR due to it not changing its expression in senescent compared to proliferating cells (González-Bermúdez et al., 2019; Hernandez-Segura et al., 2019).

With further work, the nuclear position of chromosome 10 could be a reliable marker to differentiate between quiescent and replicative senescent cells, since there are presently a range of issues with biomarkers to differentiate decisively and easily between the two non-proliferating states (Hernandez-Segura et al., 2018), a number of markers is combined to be more certain (Gorgoulis et al., 2019) and even then it is not so easy to differentiate between different types of senescence.

**FIGURE 8:**
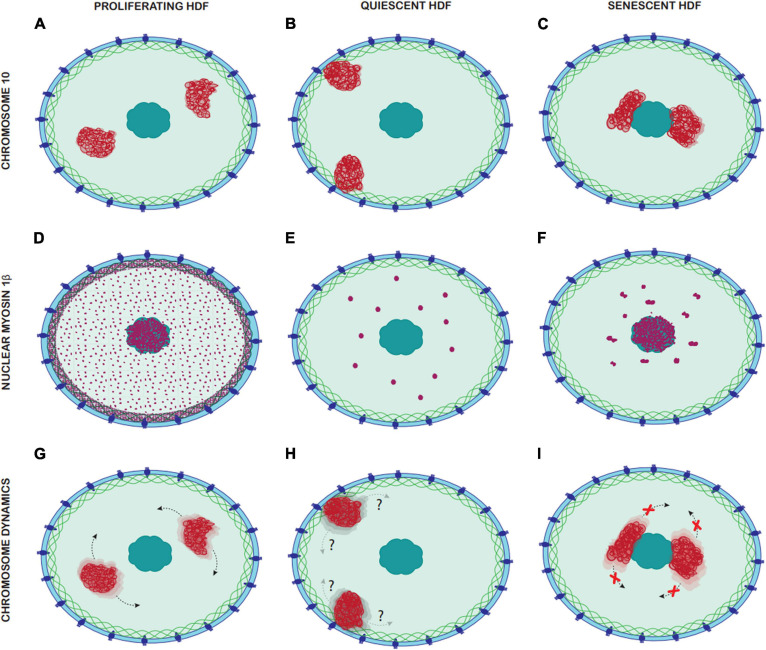
Comparison of Chromosome 10 location, nuclear myosin 1β distribution and chromosome dynamics in proliferating, quiescent, and senescent HDF. Panels **(A–C)** represent the different locations of chromosome 10 in proliferating, quiescent and senescent HDF, with chromosome 10 territories represented in red. **(A)** In proliferating HDF chromosome 10 occupies and intermediate nuclear position. **(B)** In quiescent HDF chromosomes 10 occupies a peripheral nuclear position. **(C)** In senescent HDF chromosome 10 occupies an interior nuclear position. Panels **(D–F)** represent the distribution of nuclear myosin 1β in the nucleus of proliferating, quiescent, and senescent HDF, with nuclear myosin 1β represented in purple. **(D)** In proliferating HDF, there is a dense accumulation of nuclear myosin 1β in the nuclear lamina and nucleoli, and it is also distributed homogenously through the nucleoplasm. **(E)** In quiescent HDF, nuclear myosin 1β accumulates in large spherical aggregates through the nucleoplasm. **(F)** In senescent HDF, nuclear myosin 1β accumulates in large non-spherical aggregates through the nucleoplasm but is also densely accumulated in the nucleoli. Panels **(G–I)** represent the chromosome dynamics in proliferating, quiescent and senescent HDF, with chromosome 10 territories represented in red. **(G)** In proliferative HDF, chromosome 10 can be repositioned rapidly upon a stimulus via nuclear motors. **(H)** Chromosome dynamics in quiescent HDF remains unknown. **(I)** In senescent HDF, chromosome 10 cannot be repositioned upon stimuli and thus remains in the same nuclear location.

Thus, the spatial organisation of chromosomes within interphase nuclei not only differs between various cell types (Meaburn and Misteli, 2007; Bridger et al., 2014; Sivakumar et al., 2019), but also is distinct as cells traverse from a proliferating to a non-proliferating state in their life span; thus stressing the role of this differential organisation in genome function. In addition to this, differences in organisation of NMIβ between proliferating and non-proliferating cells also suggest a plausible role of nuclear motors in chromosomal organisation within the cell nucleus (Figure 8).

Although, nuclear motor proteins have a number of roles in genome function (Venit et al., 2020), it appears that at least one of the roles NM1β plays in young proliferating cells, whereby chromosomes and genes respond to stimuli to be relocated to new non-random active locations is not functional in old cells. The nuclear distribution of NM1β is considerably different in senescent cells when compared to young proliferating cells, so a further possible biomarker candidate? Since it is highly probable that the nuclear myosins are involved in chromosome repositioning use F-actin it is pertinent to note the accumulation of G-actin in senescent cells (Kwak et al., 2004). It is highly possible that the inability to move chromatin around upon response to a stimulus in cells is in part involved in the mechanisms to prevent re-entry of senescent cells into the proliferative cell cycle. Indeed, others have predicted that once changes to genome organisation occur in senescent cells they are metastable (Chiang et al., 2019).

## Data Availability Statement

The datasets presented in this study can be found in online repositories. The names of the repository/repositories and accession number(s) can be found below: NCBI GEO; GSE164446.

## Author Contributions

IM: experimentation and design, some writing, and figures. KR: data for heat shock – 2 graphs. RP: Figure 8. MF: data for a chromosome position. KM: experimental design and some writing. IK: senescent cells and figures. CE: RNA Seq and analysis. JB: experimental design, supervision, data analysis, writing, and figures. All authors contributed to the article and approved the submitted version.

## Conflict of Interest

The authors declare that the research was conducted in the absence of any commercial or financial relationships that could be construed as a potential conflict of interest.

## Footnotes

1 http://genome.ucsc.edu/ENCODE/protocols/dataStandards/ENCODE_RNAseq_Standards_V1.0.pdf

2 http://www.bioinformatics.babraham.ac.uk/projects/seqmonk

3 https://genomics.senescence.info/cells/

4 https://genomics.senescence.info/genes/

5 https://genomics.senescence.info/cells/
