# Supplementary material for: Interphase Chromosomes in Replicative Senescence: Chromosome Positioning as a Senescence Biomarker and the Lack of Nuclear Motor-Driven Chromosome Repositioning in Senescent Cells
Source: Front Cell Dev Biol. 2021 May 24;9:640200. doi: 10.3389/fcell.2021.640200 (PMC8185894; doi:10.3389/fcell.2021.640200)
Supplement: Supplementary file 1 [file Table_1.DOCX]

**Table S1: Locations of all human chromosomes in interphase nuclei of normal primary proliferating, quiescent and senescent human dermal fibroblasts (HDFs) using 2D FISH and erosion analysis.** a) Croft et al., 1999; b) Bridger et al., 2000; c) Boyle et al., 2001; d) Meaburn et al., 2007; e) Meaburn et al., 2008; f) Mehta et al., 2010; g) Gillespie et al., 2015; h) Bikkul et al., 2019; i) Belak et al., 2020; j) Mehta et al., 2013; k) Kulashreshtha al., 2016; l) Mehta et al., 2011; m) this study.

| Chromosome No.in order of Size (Gene Density) | Proliferating HDFs | Quiescent HDFs | Senescent HDFs |
| --- | --- | --- | --- |
| 1 (2776) | **Intermediate c, j** | **Periphery f** | Intermediate m |
| 2 (1866) | **Periphery c** | **Periphery f** | Periphery m |
| 3 (1473) | **Periphery c, e** | **Periphery f** | Periphery m |
| 4 (1164) | **Periphery c, d, e** | **Periphery f** | Periphery d, m |
| 5 (1281) | **Intermediate c, e** | **Intermediate f** | Periphery m |
| 6 (1528) | **Intermediate c** | **Periphery f** | Periphery m |
| 7 (1474) | **Periphery c, e, j** | **Periphery f** | Periphery m |
| X (1344) | **Periphery c, d, e, f, g, h, j, l, m** | **Periphery f, g, l** | Periphery d, m |
| 8 (1025) | **Intermediate c** | **Periphery f** | Intermediate m |
| 9 (1207) | **Periphery c, e, j** | **Periphery f** | Periphery m |
| 10 (1094) | **Intermediate c, e, f, g, i, j, l, m** | **Periphery f, g, l** | Interior m |
| 11 (1841) | **Periphery/Intermediate c, e, k, m** | **Periphery f** | Intermed/ Periphery m |
| 12 (1355) | **Periphery c, j** | **Interior f** | Interior m |
| 13 (556) | **Periphery c, d, e** | **Interior f** | Interior d, |
| 14 (1220) | **Interior c** | **Interior f** | Interior m |
| 15 (961) | **Periphery c, k** | **Interior f** | Intermediate m |
| 16 (1108) | **Interior c, j** | **Interior f** | Intermediate m |
| 17 (1442) | **Interior c, j** | **Interior f** | Interior m |
| 18 (438) | **Periphery a, b, c, d, g, h, i, l** | **Interior b, f, g, i** | Interior b, d |
| 19 (1624) | **Interior a, b, c, j, k** | **Interior b, f** | Interior b, d |
| 20 (717) | **Interior c, j** | **Intermediate f** | Interior m |
| 22(756) | **Interior c** | **Interior f** | Interior m |
| 21 (367) | **Interior c** | **Interior f** | Interior m |
| Y (322) | Interior c | Interior f | Interior m |
