# Supplementary material for: Interphase Chromosomes in Replicative Senescence: Chromosome Positioning as a Senescence Biomarker and the Lack of Nuclear Motor-Driven Chromosome Repositioning in Senescent Cells
Source: Front Cell Dev Biol. 2021 May 24;9:640200. doi: 10.3389/fcell.2021.640200 (PMC8185894; doi:10.3389/fcell.2021.640200)

**Table S2. NMIβ distribution in control proliferating, quiescent and senescent HDFs:** Percentage of cells showing a particular pattern of NMIβ staining. Performed in triplicate on independent experiments. At least 200-500 cells counted for each replicate. Error is denoted by standard deviation.


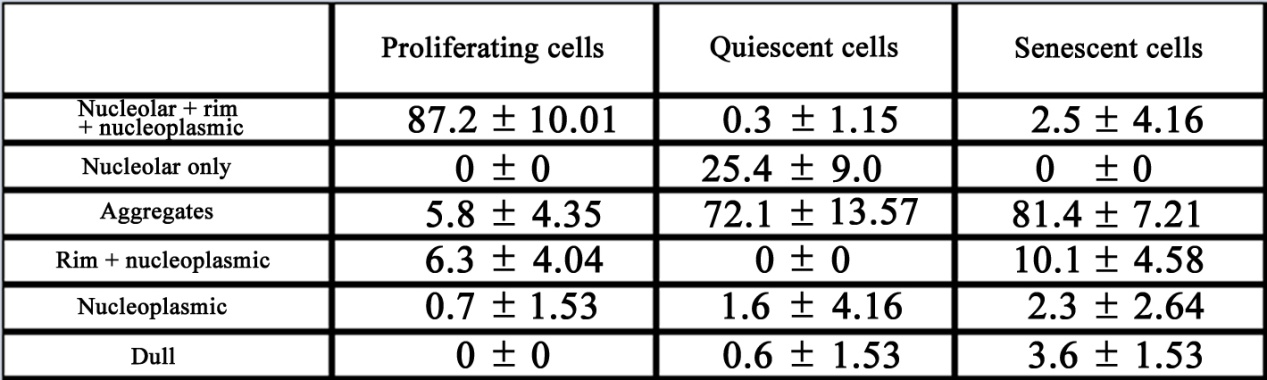

Supplement: Supplementary file 2 [file Table_2.docx]
